# O Impacto Clínico dos Sintomas Cardiovasculares na Síndrome Pós-Aguda de COVID-19

**DOI:** 10.36660/abc.20230282

**Published:** 2023-06-02

**Authors:** Giovanni Possamai Dutra, Bruno Ferraz de Oliveira Gomes

**Affiliations:** 1 Hospital Barra D’Or Rio de Janeiro RJ Brasil Hospital Barra D’Or, Rio de Janeiro, RJ – Brasil; 2 Universidade Federal do Rio de Janeiro Rio de Janeiro RJ Brasil Universidade Federal do Rio de Janeiro, Rio de Janeiro, RJ – Brasil

**Keywords:** COVID-19, Pandemia, Mortalidade, Comorbidade, Doenças Cardiovasculares, Saúde Pública, Doença Cardiopulmar

A pandemia de COVID-19 tem impactado intensamente a vida das pessoas em todo o mundo, gerando, além de alta mortalidade, alta morbidade tardia e afetando negativamente os sistemas de saúde, permanecendo um significativo desafio global na atualidade. Em fevereiro de 2023, os registros da Organização Mundial da Saúde apontam para mais de 756 milhões de casos confirmados e aproximadamente 6.845.000 mortes. Até o referido mês, foram notificados aproximadamente 36.961.000 casos no Brasil, com cerca de 698.000 mortes. Considerando os desafios substanciais relacionados à comorbidade entre doença cardíaca e COVID-19, que vão além da fase aguda da doença, são necessários mais estudos para abordar de forma abrangente a alta carga de sintomas cardiopulmonares. Essas investigações devem ser cuidadosamente planejadas, considerando custo-efetividade e benefícios clínicos para os pacientes para minimizar as desigualdades na atenção à saúde. Além disso, é essencial otimizar o cuidado social e mental dos pacientes. O envolvimento cardiopulmonar na COVID-19 continua a representar um grande desafio de saúde pública.^1 , 2^

O estudo de Kalil-Filho et al.,^3^ incluiu 480 sobreviventes após internação por COVID-19 e avaliou as características associadas à ocorrência de sintomas cardiopulmonares e, principalmente, síndrome pós-aguda de COVID-19 (PACS).^3^ Foi uma amostra com predominância de homens, obesos e com comorbidades como hipertensão, diabetes e dislipidemia. Quase um quarto dos pacientes necessitou de internação em terapia intensiva e apenas 12,2% necessitaram de suporte ventilatório mecânico. A prevalência de algum dos sintomas foi menor (32,1%) do que a encontrada na literatura, assim como a PACS relacionada a sintomas cardiopulmonares (16,3%). Notavelmente, quase dois terços da população que realizou tomografia (n=122) não demonstrou envolvimento pulmonar. Assim, observamos que a população analisada apresentou menor grau de gravidade relacionado ao COVID-19, justificando a menor prevalência de sintomas.

A síndrome pós-COVID que afeta alguns pacientes após um evento agudo de COVID-19 tem várias definições. Essa síndrome é geralmente considerada em pacientes que permanecem com sintomas após 1 a 3 meses.^4^ Os sintomas mais comuns, relatados em vários estudos, são fadiga, dispneia e distúrbios do sono.^5^ No estudo de Kalil-Filho,^3^ o sintoma mais prevalente foi o cansaço, presente em quase metade da população. Sintomas como dispneia e tosse foram menos prevalentes e podem estar associados à menor gravidade desses pacientes.

As variáveis mais associadas aos sintomas cardiopulmonares foram tempo de internação, necessidade de unidade de terapia intensiva (UTI) e ventilação mecânica, presença de polineuropatia de doença crítica e níveis de proteína C-reativa (PCR). Mahmud et al. encontraram resultados semelhantes.^6^ Neste estudo, as variáveis associadas à síndrome pós-COVID foram: sexo feminino, duração da doença, positividade do teste após 14 dias e COVID-19 grave. Em pacientes de maior gravidade, espera-se maior ativação da cascata inflamatória e indução do sistema trombótico,^7^ justificando assim o maior risco de manutenção dos sintomas a longo prazo.

O impacto dos sintomas cardiopulmonares também foi avaliado neste estudo. Pacientes sintomáticos apresentaram pior qualidade de vida e maior prevalência de ansiedade, depressão e transtorno de estresse pós-traumático (TEPT). Aiyegbusi et al.,^8^ mostraram que quase 70% dos pacientes apresentavam algum grau de limitação física após 6 meses de internação. Da mesma forma, mostrou que um quarto dos pacientes apresentava sintomas moderados a graves de TEPT. Esse diagnóstico foi mais comum em mulheres e pacientes com história de doença psiquiátrica.^8^ Ansiedade e depressão foram frequentes neste estudo, demonstrando a relevância do estudo de Kalil-Filho.^3^

Por fim, na análise multivariada, observamos que as variáveis independentes relacionadas ao diagnóstico de PACS foram sexo feminino, trombose venosa profunda, níveis de troponina e proteína C-reativa e depressão. Esses achados reforçam a importância da magnitude da resposta inflamatória e trombótica do COVID-19 no surgimento de sintomas a longo prazo.

A pandemia de COVID-19 teve um impacto significativo na saúde pública global, com alta mortalidade e morbidade tardia, incluindo a ocorrência de sintomas cardiopulmonares. Este estudo, portanto, leva à reflexão sobre a necessidade de investigações abrangentes para abordar sua alta frequência, considerando o custo-efetividade e os benefícios clínicos para os pacientes para minimizar as desigualdades na assistência à saúde. Os sintomas cardiopulmonares pós- COVID são multifatoriais e requerem uma abordagem multidisciplinar.
