# Supplementary material for: O Impacto Clínico dos Sintomas Cardiovasculares na Síndrome Pós-Aguda de COVID-19
Source: Arq Bras Cardiol. 2023 Jun 2;120(5):e20230282. [Article in Portuguese] doi: 10.36660/abc.20230282 (PMC10263397; doi:10.36660/abc.20230282)
Supplement: Translation

# Translation: The Clinical Impact of Cardiovascular Symptoms on Post-Acute COVID-19 Syndrome

**Authors:** Giovanni Possamai Dutra, Bruno Ferraz de Oliveira Gomes

**Affiliations:** 1 Brasil Hospital Barra D’Or, Rio de Janeiro, RJ – Brasil; 2 Brasil Universidade Federal do Rio de Janeiro, Rio de Janeiro, RJ – Brasil

**Keywords:** COVID-19, Pandemics, Mortality, Comorbidity, Cardiovascular Diseases, Public Health, Pulmonary Heart Disease

The COVID-19 pandemic has intensely impacted people’s lives worldwide, generating, in addition to high mortality, high late morbidity, and negatively affecting health systems, remaining a significant global challenge today. In February 2023, World Health Organization records point to more than 756 million confirmed cases and approximately 6,845,000 deaths. Until the abovementioned month, approximately 36,961,000 cases were reported in Brazil, with about 698,000 deaths. Considering the substantial challenges related to comorbidity between heart disease and COVID-19, which go beyond the acute phase of the disease, further studies are needed to address the high burden of cardiopulmonary symptoms comprehensively. These investigations must be carefully planned, considering cost-effectiveness and clinical benefits for patients, to minimize inequalities in health care. In addition, it is essential to optimize social and mental care for patients. Cardiopulmonary involvement in COVID-19 continues to pose a major public health challenge.^1 , 2^

The study by Kalil-Filho et al.^3^ included 480 survivors after hospitalization due to COVID-19 and evaluated the characteristics associated with the occurrence of cardiopulmonary symptoms and, especially, post-acute COVID-19 syndrome (PACS).^3^ It was a sample with a predominance of men, obese and with comorbidities such as hypertension, diabetes, and dyslipidemia. Almost a quarter of patients required intensive care admission, and only 12.2% required mechanical ventilation support. The prevalence of any of the symptoms was lower (32.1%) than that found in the literature, as well as PACS related to cardiopulmonary symptoms (16.3%). Notably, almost two-thirds of the population that underwent tomography (n=122) did not demonstrate pulmonary involvement. Thus, we observed that the analyzed population had a lower degree of severity related to COVID-19, justifying the lower prevalence of symptoms.

The post-COVID syndrome that affects some patients after an acute COVID-19 event has several definitions. This syndrome is generally considered in patients who remain with symptoms after 1 to 3 months.^4^ The most common symptoms, reported in several studies, are fatigue, dyspnea, and sleep disturbances.^5^ In Kalil-Filho’s study,^3^ the most prevalent symptom was tiredness, which was present in almost half of the population. Symptoms such as dyspnea and cough were less prevalent and may be associated with the lower severity of these patients.

The variables most associated with cardiopulmonary symptoms were the length of hospital stay, need for intensive care unit (ICU) and mechanical ventilation, presence of critical illness polyneuropathy, and C-reactive protein (CRP) levels. Mahmud et al. found similar results.^6^ In this study, the variables associated with the post-COVID syndrome were female gender, duration of illness, test positivity after 14 days, and severe COVID-19. In patients with greater severity, greater activation of the inflammatory cascade and induction of the thrombotic system is expected,^7^ thus justifying the greater risk of maintaining symptoms in the long term.

The impact of cardiopulmonary symptoms was also evaluated in this study. Symptomatic patients had worse quality of life and a higher prevalence of anxiety, depression, and post-traumatic stress disorder (PTSD). Aiyegbusi et al.^8^ showed that almost 70% of patients had some degree of physical limitation after 6 months of hospitalization. Likewise, it showed that a quarter of patients had moderate to severe symptoms of PTSD. This diagnosis was more common in women and patients with a history of psychiatric illness.^8^ Anxiety and depression were frequent in this study, demonstrating the relevance of Kalil-Filho’s study.^3^

Finally, in the multivariate analysis, we observed that the independent variables related to the diagnosis of PACS were female gender, deep vein thrombosis, troponin and C-reactive protein levels, and depression. These findings support the importance of the magnitude of the inflammatory and thrombotic response of COVID-19 in the emergence of symptoms in the long term.

The COVID-19 pandemic has had a significant impact on global public health, with high mortality and late morbidity, including the occurrence of cardiopulmonary symptoms. This study, therefore, leads to reflection on the need for comprehensive investigations to address its high frequency, considering the cost-effectiveness and clinical benefits for patients to minimize inequalities in health care. Post-COVID cardiopulmonary symptoms are multifactorial and require a multidisciplinary approach.
